# Editorial: Viruses and immune response in pediatric infection

**DOI:** 10.3389/fcimb.2023.1257807

**Published:** 2023-07-25

**Authors:** Jixin Yang, Jiexiong Feng

**Affiliations:** Department of Pediatric Surgery, Tongji Hospital, Tongji Medical College, Huazhong University of Science and Technology, Wuhan, China

**Keywords:** virus, child, immunity, infection, inflammation

The occurrence of many infectious diseases in children is directly related to initial viral infection, while the immune response against viral Infections is responsible for subsequent pathophysiological changes (Getts et al., 2013). Children differ greatly from adults with respect to their susceptibility to viruses and the kind of immune reaction they elicit (Prendergast et al., 2012). Infants usually represent a critical window when they are exposed to different environments, and viral infection can regulate the maturity of immunocytes and even remodel the function of their immune system (Renz and Skevaki, 2021). This means that innate immunity evolves during development from neonates through adults (Schreurs et al., 2021), for example, weaker interferon responses that may explain their increased susceptibility to viral infections. Among those, a very typical disease is biliary atresia (BA). BA has been recognized as a virus-induced autoimmune disease (Mack, 2007), in which infection by viruses, especially rotavirus, is often considered as the initiator in the pathogenesis. As is known, activation of NK cells is found to be up-regulated by inflammatory cytokines in an age-dependent fashion(Sundstrom et al., 2007). As NK cells gain increasing activation as mice age, they gain increasing cytotoxicity on rotavirus-infected cholangiocytes, which leads to persistent biliary injury and causes BA (Qiu et al., 2014). On the other hand, adult NK cells eliminate rotavirus-infected cholangiocytes shortly after infection, which prevents persistent rotavirus infection in bile ducts in this context. Besides BA, there are some other viral infectious diseases in children with a similar pattern of immunocyte maturation. Therefore, the special relationship between immunocyte maturation and viral infection in children requires in-depth research in future.

In this thematic Research Topic, 5 original researches of high-quality are presented to readers. The authors have studied the characteristics, treatment methods, and evidence of immune function changes in children after viral infection from different perspectives, and have pointed out future research hotspots to readers.

Although COVID-19 has become a research hotspot in the past three years, the reasons why the incidence of COVID-19 infection in children is lower than that in adults still remain unclear. Especially, very little is known about the T cell response in children with COVID-19. Russo et al. offer a novel point of view that the development of different CD4^+^ T cell profiles in the course of pediatric COVID-19 and manifested the possible mechanism. They showed imbalance in the CD4^+^ T cell compartment during pediatric COVID-19 and suggest the involvement of extracellular ATP during its induction. The researchers also point out prospectively that the mechanisms by which the purinergic signaling modulates the adaptive immune response against SARS-CoV-2 in children should be focused on. It is also necessary to define whether extracellular ATP levels could be a biomarker of severity in this disease.

Multisystem inflammatory syndrome (MIS) is a rare but very severe condition associated with COVID-19 in which different organs become inflamed, including the heart, lungs, kidneys, brain, skin, eyes, intestines, liver et al. MIS may affect children (MIS-C) or adults (MIS-A). Reis et al. performed whole exome sequencing in patients with MIS-C and further analyzed its characteristics and association with MIS-A. Their team identified the presence of 50 variants in 38 different genes, including those not previously reported (KMT2D, CFB, and PRF1, APOL1, TNFRSF13B, and G6PD). By highlighting the biological processes identified from the genes, the research helps understand which pathways are potentially compromised, opening the way for a new diagnostic and treatment system in the follow-up of patients with MIS-C.

Influenza B virus is a segmented negative-strand RNA virus, which often leads to seasonal epidemics of disease in people. It dominates influenza seasons and causes severe disease, particularly in children and adolescents, who have a higher mortality rate. Therefore, it poses a serious threat to public health and health. Ma et al. conducted a retrospective cohort study including Chinese children with influenza B virus pneumonia. They revealed a relationship between the body’s immune status and disease severity after influenza B virus infection. A very important finding is that the decreased number of CD8^+^ T cells and NK cells can be used as independent risk factors for predicting the severity of influenza B virus pneumonia in children. Therefore, in addition to white blood cell count and CRP, it is necessary to monitor the lymphocyte subsets of children with influenza B virus pneumonia in the early stage.

Epstein-Barr virus (EBV) is a member of the herpes virus family. People may become infected with EBV in childhood and EBV infections in children do not usually cause symptoms, or the symptoms are not distinguishable from other mild, brief childhood illnesses. Ye et al. investigated the epidemiology and infectious characteristics of EBV infection among children in Shanghai, China from 2017 to 2022. By retrospectively collecting and analyzing demographic information, diagnosis, laboratory tests and complications, they pointed out that when co-infected with EBV to other microbial pathogens, which is commonly seen, more EBV viral loading and higher level of inflammatory cytokines may present in these patients.

Acute respiratory infections (ARI) are the leading cause of death among patients under five years old. It was estimated to be responsible for around 2 million childhood deaths globally. In Li et al’s. meta-analyses and trial sequential analysis, they show evidence that dynamic procalcitonin (PCT) level is considered to be a specific infection-related biological marker to guide the rational use of antibiotics when treating ARI. They analyzed 4 Randomized Controlled Trials including 1313 patients with acute respiratory infection, and stated that PCT-guided antibiotic therapy was associated with significantly shorter length of antibiotic therapy compared with the control group. Additionally, a decreased rate of antibiotic adverse events was observed. Hence, for pediatrics with acute respiratory infection, PCT-guided treatment has the potential to decrease antibiotics exposure.

In summary, the editors of this Research Topic believe that this series of articles have provided us with Evidence-based research of viral infection related diseases in children, and expanded our understanding of the different immune responses between children and adults after viral infection. We hope that our target readers will start research in the area of discovering potential prognostic markers and propose more possible novel therapeutic methods in future.

## Author contributions

JY drafted the original manuscript, and a. JF conceptualized manuscript, performed review and editing the manuscript. Both authors agree to be accountable for the content of the work. All authors contributed to the article and approved the submitted version.

## References

[B1] Getts D. R. Chastain E. M. Terry R. L. Miller S. D. (2013). Virus infection, antiviral immunity, and autoimmunity. Immunol. Rev. 255 (1), 197–209. doi: 10.1111/imr.12091 23947356 PMC3971377

[B2] Mack C. L. (2007). The pathogenesis of biliary atresia: evidence for a virus-induced autoimmune disease. Semin. Liver Dis. 27 (3), 233–242. doi: 10.1055/s-2007-985068 17682970 PMC3796656

[B3] Prendergast A. J. Klenerman P. Goulder P. J. (2012). The impact of differential antiviral immunity in children and adults. Nat. Rev. Immunol. 12 (9), 636–648. doi: 10.1038/nri3277 22918466

[B4] Qiu Y. Yang J. Wang W. Zhao W. Peng F. Xiang Y.. (2014). HMGB1-promoted and TLR2/4-dependent NK cell maturation and activation take part in rotavirus-induced murine biliary atresia. PloS Pathog. 10 (3), e1004011. doi: 10.1371/journal.ppat.1004011 24651485 PMC3961347

[B5] Renz H. Skevaki C. (2021). Early life microbial exposures and allergy risks: opportunities for prevention. Nat. Rev. Immunol. 21 (3), 177–191. doi: 10.1038/s41577-020-00420-y 32918062

[B6] Schreurs R. Sagebiel A. F. Steinert F. L. Highton A. J. Klarenbeek P. L. Drewniak A.. (2021). Intestinal CD8(+) T cell responses are abundantly induced early in human development but show impaired cytotoxic effector capacities. Mucosal Immunol. 14 (3), 605–614. doi: 10.1038/s41385-021-00382-x 33772147 PMC8075922

[B7] Sundstrom Y. Nilsson C. Lilja G. Karre K. Troye-Blomberg M. Berg L. (2007). The expression of human natural killer cell receptors in early life. Scand. J. Immunol. 66 (2-3), 335–344. doi: 10.1111/j.1365-3083.2007.01980.x 17635811

